# Dyadic Interdependence in Non-spousal Caregiving Dyads’ Wellbeing: A Systematic Review

**DOI:** 10.3389/fpsyg.2022.882389

**Published:** 2022-04-29

**Authors:** Giulia Ferraris, Srishti Dang, Joanne Woodford, Mariët Hagedoorn

**Affiliations:** ^1^Department of Health Psychology, University Medical Center Groningen, University of Groningen, Groningen, Netherlands; ^2^Healthcare Sciences and e-Health, Department of Women’s and Children’s Health, Uppsala University, Uppsala, Sweden

**Keywords:** interdependence, non-spousal dyads, caregiving, intrapersonal, interpersonal, wellbeing

## Abstract

Caregiving dyads (i.e., an informal caregiver and a care recipient) work as an interdependent emotional system, whereby it is assumed that what happens to one member of the dyad essentially happens to the other. For example, both members of the dyad are involved in care giving and care receiving experiences and therefore major life events, such as a serious illness affect the dyad and not only the individual. Consequently, informal caregiving may be considered an example of dyadic interdependence, which is “the process by which interacting people influence one another’s experience.” This systematic review aimed to synthesize studies of dyadic interdependence, specifically in non-spousal caregiving dyads (e.g., adult children—parents, siblings, other relatives, or friends). Electronic databases (PsycINFO, Pubmed, and CINAHL) were systematically searched for dyadic studies reporting on interdependence in the emotional and relational wellbeing of non-spousal caregiving dyads. A total of 239 full-text studies were reviewed, of which 14 quantitative and qualitative studies met the inclusion criteria with a majority of dyads consisting of adult daughters caring for their older mothers. A narrative synthesis suggested mutual influences between non-spousal caregiving dyad members based on: (1) associations between intrapersonal (e.g., psychological functioning) and interpersonal (e.g., relationship processes) variables and emotional and relational wellbeing of the dyad; (2) associations between care context variables (e.g., socio-demographics and care tasks) and emotional and relational wellbeing of the dyad; and (3) patterns of covariation between caregivers’ and care recipients’ wellbeing. Evidence supporting dyadic interdependence among non-spousal caregiving dyads shed light on the ways dyad members influence each other’s wellbeing while providing and receiving care (e.g., via the exchange of support). Future studies investigating mutual influences in dyads, should differentiate subsamples of caregivers based on relationship type, and adopt dyadic and longitudinal designs.

**Systematic Review Registration:**

[https://www.crd.york.ac.uk/prospero/#recordDetails], identifier [CRD42021213147].

## Introduction

Informal care arises from a communal relationship between an informal caregiver (hereafter referred to as caregiver) and the person in need of care (i.e., the care recipient). Evidence suggests that caregiver and care recipient wellbeing is mutually interconnected, and adaptation to disease or aging often involves both members of the caregiving dyad (Meyler et al., 2007; Kelley et al., 2019; Varner et al., 2019). Using Cook and Kenny’s definition, “there is interdependence in a relationship when one person’s emotion, cognition, or behavior affects the emotion, cognition, or behavior of a partner” (Cook and Kenny, 2005, p. 101). For instance, caregivers’ psychological wellbeing might be profoundly influenced by reactions and emotional experiences of care recipients and, in turn, care recipients’ adjustment to illness might be influenced by the way they perceive caregivers (Hagedoorn et al., 2011a; Fife et al., 2013; Revenson et al., 2016). Indeed, research suggests caregiver and care recipient intrapersonal (e.g., psychological functioning) and interpersonal (e.g., relationship processes) variables interact with each other and contribute to individual (i.e., emotional) and dyadic (i.e., relational) adjustment to illness (Karademas, 2021). Intrapersonal variables refer to individual-level characteristics, for example, attitudes and beliefs, psychological distress, and personality traits, whereas interpersonal variables refer to dyadic-level interactions and relationship processes that occur between at least two people (Rusbult and Van Lange, 2003; Van Lange and Rusbult, 2012; Karademas, 2021).

To varying degrees, illnesses affect caregiving dyads as a unit, rather than isolated individuals, resulting in dyad members having mutual impact on each other regarding quality of life, psychological health, and relationship functioning (Revenson et al., 2016). These processes of mutual influences may change over time and might be affected also by the care context in which caregivers and care recipients are both embedded (e.g., culture, illness condition, care tasks, perceptions and evaluation of the broader social environment) (Berg and Upchurch, 2007; Revenson et al., 2016). In other words, while providing and receiving care, caregivers’ and care recipients’ shared psychosocial context may mutually shape emotional outcomes as well as relational adjustment (Van Lange and Rusbult, 2012).

This kind of dyadic interdependence and connectedness is usually seen as a core defining feature of couples, given the strong emotional and physical closeness that often characterizes romantic partners or spouses (hereafter referred to collectively as spouses). Indeed, reviews of research on spouses in the illness context support dyadic interdependence within couples. For example, cancer experiences have been found to be highly interdependent between spouses (Hodges et al., 2005; Hagedoorn et al., 2008) and a recent systematic review suggests an interdependence of physical and psychological morbidity among patients with cancer and their family caregivers (Streck et al., 2020). Although this recent review includes both spousal and non-spousal caregiving dyads, the majority of studies examine only spousal dyads. A point that is often neglected is that dyadic interdependence can also occur within other relationships (e.g., parents and adult children, other relatives or friends) where there is an emotional bond and some degree of closeness (Clark and Mills, 2012; Le et al., 2018). However, currently little is known about how the wellbeing of one dyad member depends on the other member within non-spousal relationships. Given that caregiving experiences may be different based on the type of relationship between caregivers and care recipients (i.e., being a spouse or another family member), generalization of spousal literature might not always be appropriate (Sheehan and Donorfio, 1999; Greenwood et al., 2008; Kwak et al., 2012). Indeed, a review comparing spousal caregivers and adult children/children in law found a number of differences between the two caregiver groups (Pinquart and Sörensen, 2011). For example, spouses usually provide more support to their loved ones, but report fewer care recipient behavior problems than adult-child caregivers. Conversely, adult children report fewer depressive symptoms and higher levels of psychological wellbeing than spousal caregivers (Pinquart and Sörensen, 2011). Similarly, in a longitudinal study, spousal caregivers reported more mental health problems, physical health impairments, and difficulties in combining daily activities with care tasks, compared to adult-child caregivers. However, adult children caregivers reported more distress and burden when intensity of care was higher in terms of time investment (Oldenkamp et al., 2016). Although these studies demonstrate how different caregiver groups respond to and are influenced by the caregiving experience, less is currently known about how non-spousal caregiving dyads may mutually influence each other’s wellbeing. As such, there is a need to review the existing caregiving literature with regard to the role of dyadic interdependence in the emotional and relational wellbeing of non-spousal dyads.

The role of dyadic interdependence within non-spousal dyads is important for a number of additional reasons. First, in most cases, informal care is almost equally directed toward spouses and older parents, with on average 36% of informal caregivers caring for their spouse (spousal caregivers) and 32% caring for a parent (adult-child caregivers). There is also a relatively high proportion of caregivers who report helping a friend or a neighbor (18%) or taking care of other relatives such as brothers/sisters or aunts/uncles (18%) (Colombo et al., 2011). Moreover, the rate of older people (i.e., 65 years and above) with long term care needs is expected to almost double from 17% in 2010 to 30% in 2060 across Europe, resulting in an increased need for the provision of informal care and a growing number of family members (e.g., adult children) and friends will be called upon to fulfill the role of caregiver (European Commission, 2020). Lastly, taking into account the unique type of caregiver-care recipient relationship (i.e., spousal caregivers or non-spousal caregivers) may help provide more tailored interventions based on the diversity of caregiving dyad relationships (Braun et al., 2009).

Theoretical frameworks such as dyadic coping models (see Falconier and Kuhn, 2019, for more detail), equity theory (Van Yperen and Buunk, 1990) and interdependence theory (Rusbult and Van Lange, 2003; Van Lange and Rusbult, 2012) have been successfully applied to health studies demonstrating the importance of interactions, mutual dependency, and dyadic influences in the illness context. Given the explorative aim of this systematic review, interdependence theory constituted a theoretical guide to establish the connection between dyad members. Interdependence theory is an important framework for understanding social relationships as it concerns how dyad members influence each other’s outcomes. Three relevant dimensions of interdependence were considered: (a) the “level of dependence” which describes the impact of each dyad member on the outcomes of the other one (i.e., when one’s variable is associated with or influences the other’s outcomes); (b) the “structure” that is the shared context of a given situation where the dyad members interact; and (c) the “covariation of interests,” which describes the degree to which dyad members outcomes correspond to each other (i.e., when one’s variable tends to increase or decrease in value also the corresponding values of the other one’s variable tend to increase or decrease) (Van Lange and Rusbult, 2012).

This systematic review aimed to synthesize three levels of evidence for dyadic interdependence in emotional and relational wellbeing of non-spousal caregiving dyads (e.g., adult children—parents, siblings etc.): (1) first level of evidence for interdependence is whether some characteristics of one member of the dyad are associated with the wellbeing of the other dyad member, and whether the interactions between the two dyad members are associated with the wellbeing of both; (2) second level of evidence for interdependence is in terms of associations between care context variables and wellbeing in both dyad members; and (3) third level of evidence for interdependence is about patterns of covariation between dyads members, that is whether both dyad members report similar wellbeing and emotional states.

## Methods

The current review was conducted in accordance with the Preferred Reporting Items for Systematic Reviews and Meta-Analyses (PRISMA) guidelines (Page et al., 2021; see Supplementary Material 1). Moreover, it was registered in PROSPERO international Prospective Register of Systematic Reviews database in advance of the review being conducted (registration number ID = CRD42020215259). The scope of the review was determined using the PICOS tool where possible (participants, outcomes, and study design) (see Supplementary Material 2; Methley et al., 2014).

### Search Strategy

A search of three electronic databases (PsycINFO, Pubmed, and CINAHL) was conducted from database inception to December 2021. The search strategy was developed in consultation with a librarian at the University Medical Center of Groningen and was reviewed following the PRESS peer review guidelines (McGowan et al., 2016). The search strategy was designed in PsycINFO and then translated to the appropriate MESH/thesaurus terms and formats for the other databases (Supplementary Material 2). The search was restricted to studies published in English in peer−reviewed journals. Key population-related search terms included “family,” “adult child,” “parent,” “carer,” or “caregiver.” The search also included the following key terms related to the phenomena of interest: “interpersonal relations,” “communication,” “mutuality,” “interpersonal influences,” “dyadic coping,” “responsiveness,” “interdependence,” “congruence,” “family processes,” “relationship change,” and to the outcomes: “depression,” “anxiety,” “stress,” “quality of life,” “wellbeing,” “burden,” and “relationship satisfaction.” The complete search strategy is detailed in Supplementary Material 2. In addition to the search, backward and forward reference searching of included studies was conducted to identify any study not retrieved through database searching.

### Eligibility Criteria

#### Participants

Adult (≥18 years old) non-spousal caregiver and adult care recipient dyads. Care recipients were community dwelling and had a chronic illness, physical disability, or frailty due to aging. Studies including spousal caregiving dyads in an intimate and romantic relationship, whereby the data was not reported separately for non-spousal dyads, were excluded.

#### Outcomes

Studies were included if predictor and/or outcome variables were measured for both dyad members and if they reported intrapersonal, interpersonal, or context variables possibly impacting or associated with the wellbeing of dyad members. Intrapersonal variables included levels of distress, psychological functioning, and personality traits. Interpersonal variables included communication patterns, exchange of support, dyadic interactions. Care context variables included: socio-demographic variables, health status, care needs, and care tasks. Studies were also included if they reported on covariations between caregivers’ and care recipients’ wellbeing.

#### Study Design

Qualitative (e.g., semi-structured interviews), quantitative (e.g., cross-sectional and longitudinal designs) and mixed methods designs were eligible for inclusion. Given the observational nature of this systematic review, randomized controlled trials, quasi-experimental, and case studies were excluded.

### Selection of Studies

Study selection was performed in two phases by two independent reviewers (GF, SD) who referred to a third reviewer (MH) if an agreement about inclusion could not be reached. The first reviewer (GF) screened all the studies, while the second reviewer screened 10% of the total amount of studies (Gough et al., 2012). In the first phase, titles and abstracts retrieved from searches were screened. In the second phase, reviewers screened potential eligible studies for final inclusion based on full-paper checks. The online screening software Rayyan facilitated the study selection process (Ouzzani et al., 2016).

### Data Extraction

Data from included studies were extracted with data entered in Microsoft Excel (2016), using a data extraction form developed for this review based on the Cochrane data collection form for intervention reviews on randomized controlled trials (RCTs) and non-RCTs (Li et al., 2021). The first reviewer (GF) extracted data from all included studies, while the second reviewer (SD) independently extracted data from 30% of the studies. Any conflict or doubt that emerged was resolved by discussion. The following information was extracted from each included study: author, year, and country; study design; study objectives; the unit of analysis (i.e., number and type of dyads), care recipient’s health condition; a general description of participants including age and gender; predictors/correlates; outcome measures; levels of evidence for dyadic interdependence (i.e., dyad members’ outcomes associated with intrapersonal, interpersonal, and care context variables, and covariations between dyad members).

### Assessment of Quality

Assessment of study quality was conducted using the Mixed Methods Appraisal Tool (MMAT) (Hong et al., 2018). This critical appraisal tool permits to assess the methodological quality of five categories of studies: qualitative research, randomized controlled trials, non-randomized studies, quantitative descriptive studies, and mixed methods studies. Depending on the research design, each included study was evaluated according to five different questions. Two investigators (GF and SD) assessed the included papers independently according to the MMAT, discrepancies were discussed, and consensus was reached. Studies were assigned an overall quality score ranging from (0/5) to (5/5) based on methodological quality criteria.

### Data Synthesis

A small number of dyadic studies reporting on dyadic interdependence were found (*n* = 14), and given the different theoretical conceptualizations and the heterogeneity in the sample population, a quantitative analysis was not considered appropriate (Li et al., 2021) and a narrative synthesis approach was adopted.

Narrative methods are often used to summarize and explain findings from multiple studies adopting a textual approach to “tell the story” of the findings (Popay et al., 2006). Our narrative synthesis included four steps. In the first step *descriptive paragraphs* on each included study were systematically produced with the same information in the same order for all the studies (e.g., aims, interpersonal/intrapersonal/care context/covariations variables, design and analysis, significant findings regarding interdependence between dyad members). The second step included *tabulation*: a general table was created to better define the predictors, outcomes, or correlates. The third step consisted of organizing the included studies into thematic *groups* depending on patterns (similarities/differences) within and across these studies. Studies were clustered according to the characteristics in the data extraction table (i.e., findings on levels of interdependence). Finally, the fourth step included *concept mapping* that is creating diagrams or flow charts to visually represent the relationships being explored. This technique aimed at linking evidence extracted from the included studies, highlighting key concepts such as dyadic interdependence between caregivers and care recipients and representing relationships between these factors (i.e., dyad members’ outcomes associated with intrapersonal, interpersonal, care context variables, and covariations between dyad members) (Popay et al., 2006; Gough et al., 2012).

## Results

### Selection of Studies

**FIGURE 1:**
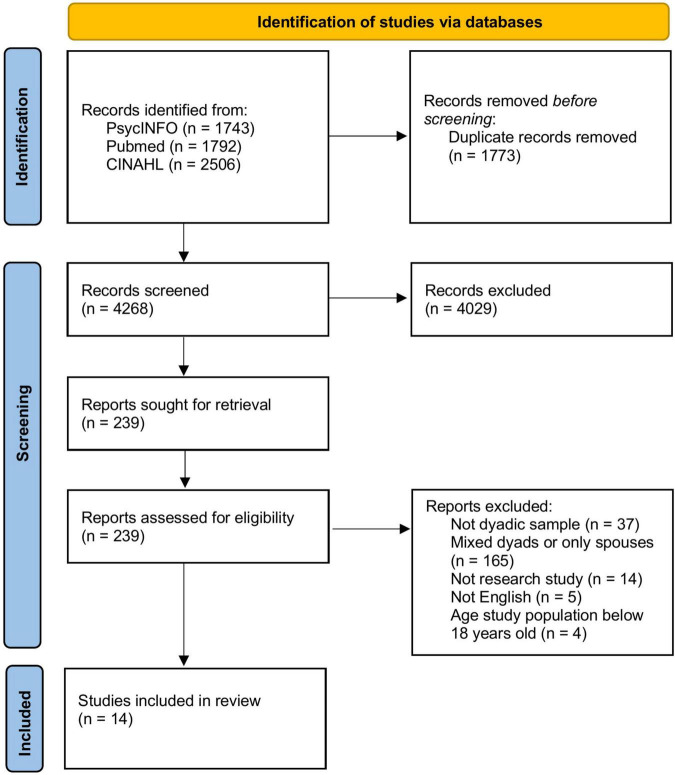
PRISMA (Preferred Reporting Items for Systematic Reviews and Meta-Analyses) flow diagram of study selection process.

The initial search identified 6,041 studies. After removing duplicates using EndNote, 4,308 studies were screened. Following title and abstract screening, the full-text of 239 studies were screened, resulting in 14 studies suitable for inclusion. Figure 1 illustrates the process of inclusion and exclusion of the studies (Page et al., 2021).

**FIGURE 2:**
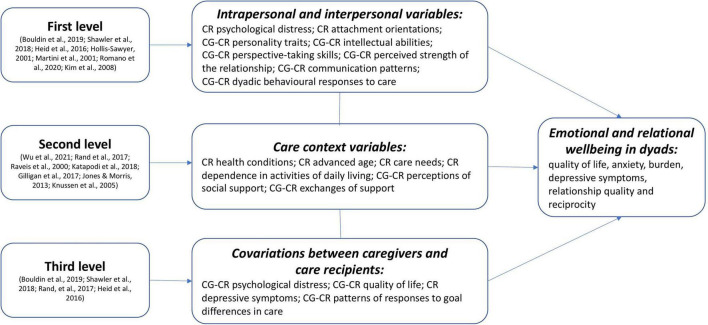
Diagram representing included studies on the three levels of evidence for dyadic interdependence.

### Study Characteristics

The main characteristics of the included studies (*n* = 14), 11 quantitative (8 cross-sectional, 3 longitudinal), two qualitative, and one mixed-method, are presented in Table 1. Eight studies were conducted in the United States, three in the United Kingdom (UK), one in Canada, one in Australia, and one in China. Sample sizes ranged from 10 to 264 dyads; two studies reported also on triads (*n* = 121; *n* = 369) (Gilligan et al., 2017; Katapodi et al., 2018). Type of relationship between caregivers and care recipients varied. In two studies (one qualitative and one quantitative) adult-children were the care recipients (e.g., presenting stroke and mixed health conditions) receiving care from parents and healthy siblings (Jones and Morris, 2013; Gilligan et al., 2017). In the other studies, populations included older parents or grandparents receiving care from younger relatives or friends, with 42% of the studies (6/14) focusing on adult-daughter caregivers (Raveis et al., 2000; Hollis-Sawyer, 2001; Martini et al., 2001; Kim et al., 2008; Heid et al., 2016; Shawler et al., 2018).

**TABLE 1:** Characteristics of included studies.

Author year country	Study design	Study objective (s)	Subjects’ description: sample size mean age CG M (SD) CR M (SD)	Care recipient’s health condition	Predictors/correlates	Outcome measures	Key findings	Levels of evidence for interdependence
Bouldin et al. (2019); United States	Cross-sectional survey study	To identify groups of Heart Failure (HF) patients and their informal caregivers and to compare how these groups agree on the patients’ depressive symptoms	*N* = 201 dyads CGs: relatives (daughters, sons, siblings or friends; 69% female), mean age = 46.3 (12.9) CRs: relatives (99% male), mean age = 68.3 (10.4)	Heart failure	CGs and CRs: Relationship characteristics (17 items about level of closeness and frequency of negative emotions during dyadic interactions) Communication (11 items about frequency and type of interactions)	CGs and CRs: CRs’ depressive symptoms (CES-D 10) Dyads’ agreement on patient depressive symptoms (Dyadic scores of CES-D-10)	Four different groups of dyads were identified based on relationship and communication between the dyad members: *Collaborative (n* = *102, 51%); Avoidant (n* = *33, 16%); Distant (n* = *35, 17%); Antagonistic (n* = *31, 16%)*. 1. Dyads’ relationship and communication characteristics were related to CRs’ depressive symptoms: dyads characterized by more positive interactions or fewer interactions *(12% of Collaborative dyads and 6% of Distant)* perceived fewer depressive symptoms in CRs (CES-D > 10). 2. CGs’ ratings of CRs’ depressive symptoms (CES-D) were moderately similar to CRs’ own self-assessments *(k* = *0.18).* Concordance and correlation between CG and CR were higher in Distant *(k* = *0.44; r* = *0.39)* and Collaborative *(k* = *0–19; r* = *0.32)* dyads.	1. Associations between CR/CG interpersonal variables (relationship characteristics and communication patterns) and CG/CR emotional wellbeing (CRs’ depressive symptoms perceived by both CG and CR) 2. CG and CR in collaborative and distant dyads showed correlations on CRs’ depressive symptoms
Heid et al. (2016); United States	Qualitative semi-structured interviews	To investigate the process by which older adults influence their care in families, the way the daughter caregivers respond to such influence and the disagreements in care between older adults and caregivers	*N* = 10 dyads CGs: adult daughters, mean age = 51.20 (10.10) CRs: parents, mean age = 79.20 (9.09)	Older adults	N/A	Dyadic behaviors responses to navigating goal differences in care	1. Findings highlighted how CGs and CRs resolve conflicts when there are differences in their care goals: CGs more often reason with their CRs, while CRs walk away from the situation or “let go” of CG’s requests. If the CR lets go of the request, the CG’s goal is met. However, when the CR continues to act on goal, is the CG the one who let go of the request, letting the CR’s goal met. 2. CGs and CRs described similar response strategies to the conflict (e.g., let go or brainstorm a new solution).	1. Associations between CR/CG interpersonal variables (dyadic behaviors responses to goal differences in care) and CG/CR emotional wellbeing (reciprocity) 2. CG and CR reported similar patterns of responses to goal differences in care
Hollis-Sawyer (2001); United States	Cross-sectional; Mixed method study	To explore the factors (individual differences and relationship role-related factors) that could predict the positive, growth-oriented responses to the caregiving experience for both caregiving daughters and care-receiving mothers	*N* = 122 dyads CGs: adult daughters, age range = 18–60 CRs: mothers, age range = 57–90	Older adults with physical impairments	CGs and CRs: Perceived family role changes (11 close-ended items and 5 open-ended items) Personality traits (NEO FFI-Form S Scale) Intellectual abilities (Culture Fair Intelligences Matrices Scale 3)	CGs and CRs: Degree of positiveness in caregiving pair: personal growth (3 close-ended items and 9 open-ended items); role congruency (4 open-ended items)	Some CRs’/CGs’ individual-difference factors (e.g., CR fluid intellectual ability *B* = *0.25 p ≤ 0.01*, CR openness in personality *B* = *0.54 p ≤ 0.01* and CG neuroticism *(B* = −*0.44 p ≤ 0.01)* were associated with the caregiving relationship (i.e., perceived positiveness in the relationship) for both the dyad members.	Associations between CR/CG intrapersonal variables (fluid intellectual ability, CR openness in personality and CG neuroticism) and the CG-CR relational wellbeing (quality of relationship)
Kim et al. (2008); United States	Cross-sectional survey study	To investigate the effect of (dis)similarity in psychological distress between mothers with cancer and their caregiving adult daughters on each person’s quality of life	*N* = 98 dyads CGs: adult daughters, mean age = 40.76 (11.74) CRs: mothers, mean age = 67.12 (12.01)	Cancer	CGs and CRs: Socio-demographics Psychological Distress (POMS-SF)	CGs and CRs: Quality of life (MOS SF-36 or MOS SF-12)	1.CRs’ greater distress was associated with CGs’ worsen mental health (quality of life domain), but not vice versa; (partner effects: *B* = *0.14 p ≤ 0.05)*. 2. Significant moderate covariations were found between CGs and CRs psychological distress *(r* = *0.27 p ≤ 0.05)*.	1. Associations between CR intrapersonal variables (psychological distress) and CG emotional wellbeing (quality of life) 2. CG and CR showed similarity in the levels of psychological distress
Martini et al. (2001); Canada	Cross-sectional survey study	To examine how feelings of interpersonal control, perspective taking, and attributions are related to satisfaction with supportive help given to older mothers by their adult daughters	*N* = 44 dyads CGs: adult daughters, mean age = 44 (8.76) CRs: mothers, mean age = 73 (7.71)	Aging	CGs and CRs: Interpersonal control (ISOC) Perspective taking abilities (SDPT) Attributions (Helping Attributions Scale)	CGs and CRs: Satisfaction with the helping relationship	CGs’ and CRs’ feelings of interpersonal control and perceptual accuracy (i.e., understanding of the partners’ thoughts) were associated with the other partners’ satisfaction with the helping relationship *[CRs’ predictors on CGs’ relationship satisfaction: F(4, 34)* = *20.35, p ≤ 0.01; CGs’ predictors on CRs’ relationship satisfaction:* *F(4, 32)* = *3.56, p ≤ 0.05]*.	Associations between CR and CG interpersonal variables (interpersonal control and perceptual accuracy) and the CG-CR relational wellbeing (quality of relationship)
Romano et al. (2020); Australia	Cross-sectional survey study	To investigate how caregiver attachment insecurity in combination with care recipient attachment insecurity exacerbates caregiver burden	*N* = 70 dyads CGs: adult children (17 men, 53 women), mean age = 51.1 (0.9) CRs: older parents (18 men, 52 women), mean age = 80.4 (7.8)	Aging	CGs and CRs: Adult Familial Attachment Scale	CGs: Caregiver burden (ZBI)	Care recipient attachment anxiety in pair with caregiver avoidant attachment were significantly and positively associated with caregiver burden (*B* = *0.29 (1.54), p ≤ 0.01)*.	Associations between CR/CG intrapersonal variables (attachment orientation) and CG emotional wellbeing (burden)
Shawler et al. (2018); United States	Longitudinal survey study	To test the impact of the quality of the mother-daughter relationship, inner strength, and perceived control on hypertension (HTN) self- management and health related quality of life (HRQOL) for both members of the dyad	*N* = 51 dyads at the baseline *N* = 46 dyads at follow-up CGs: adult daughters, mean age = 52.5 CRs: mothers, mean age = 78.1	Hypertension	CGs and CRs: Perceived inner strength of the relationship (ISQ) Perceived control on the illness (CAS-R; CAS-Family).	CGs and CRs: HTN self-management behaviors (Blood Pressure; Hill-Bone Scale) HRQOL (SF-36)	CG’s/CRs’ perceived strength of the relationship increased their own quality of life: mothers *(b* = *0.33, p* = *0.049)* and daughters *(b* = *0.65, p* = *0.002)* over time; CRs’ perceived strength of the relationship reduced CGs’ emotional problems *(b* = *−1.22, p* = *0.007)*.	Associations between CR/CG interpersonal variables (perceived strength of the relationship) and CG/CR emotional wellbeing (quality of life)
Gilligan et al. (2017); United States	Longitudinal survey study (second wave from the Within-Family Differences Study-II)	To explore whether children’s serious health conditions affected the flow of expressive and instrumental support between mothers and both the offspring with health conditions and other offspring in the family	*N* = 369 triads CGs: mothers, mean age = 77.8 (3.2); siblings: mean age = 49.4 (5.8) CRs: adult children (*N* = 1,338; 20% of them with a serious health condition; 67.8% female; 32,2% male), mean age = 49.4 (5.8)	Various health conditions	CGs and CRs: Socio-demographics Clinical information	CGs and CRs: Items about type (expressive and instrumental) and frequency of provided and received support	CRs’ illnesses affected the interpersonal and intergenerational different patterns of family support: 1. mothers were more likely to provide expressive *(B* = *1.21 (0.14) p ≤ 0.01)* and instrumental *(B* = *0.87 (0.16) p ≤ 0.01)* support to their adult children with health conditions (CR) than to their children without health concerns (secondary CG); 2. mothers (CG) with a higher proportion of children with health conditions (CR) were more likely to receive expressive support *(B* = *0.22 (0.08) p ≤ 0.01)* from children without health conditions (secondary CG).	Associations between CR intrapersonal variables (health conditions) and CG-CR relational wellbeing (reciprocity in social support)
Jones and Morris (2013); United Kingdom	Qualitative semi-structured interviews	To explore the experiences of adult stroke survivors and their parent caregivers	*N* = 1 dyad and 5 triads CGs: parents (6 mothers and 5 fathers), mean age = 65.54 CRs: adult children, mean age = 36.33	Stroke	N/A	4 themes: 1. emotional turmoil 2. significance of parents 3. negotiating independence vs. dependence 4. changed relationship	The findings highlighted specific features of the parents-young adult survivors’ relationship: some of them reported detrimental impact between CGs and CRs due to the illness, other reported couple problems between the parents and others experienced positive outcomes such as a sense of growth.	Associations between CR intrapersonal variable (health condition) and CG-CR relational wellbeing (quality of relationship)
Katapodi et al. (2018); United States	Cross-sectional survey study	To describe family support in young breast cancer survivors (YBCS) and their relatives; identify demographic, clinical and psychosocial characteristics as predictors of family support; and determine the interdependence of support in young breast cancer survivors’ relative’s family units	*N* = 189 dyads and 121 triads CGs: first/second degree relatives (*N* = 431), mean age = 43.4 (11.9) CRs: YBCS, (*N* = 310), mean age = 51.4 (5.8)	Breast cancer	CGs and CRs: Socio-demographics Clinical characteristics Cost related lack of access to care Anxiety and depression Perceived Breast Cancer Risk CRs: Fear of Cancer Recurrence (CARS) Self-efficacy after cancer	CGs and CRs: Family support (MIS; Family Support in Illness Scale; FHI)	CRs’ depressive symptoms, prior diagnosis, were associated with lower CGs’ perceived family support *(B* = *−0.369 p ≤ 0.05)*; CRs’ older age and higher self-efficacy were associated with CGs’ higher family support *(B* = *0.032 p ≤ 0.05; B* = *0.116 p ≤ 0.05)*; CGs’ higher income *(B* = *0.019 p ≤ 0.05)* was associated with CGs’ higher perceived support.	Associations between CR and CG intrapersonal variables (CR: depressive symptoms, older age, higher self-efficacy; CG: income) and CG-CR relational wellbeing (reciprocity in social support)
Knussen et al. (2005); United Kingdom	Longitudinal survey study	To determine whether deterioration in family relationships could be explained by baseline values and changes in subjective and/or objective primary stressors	*N* = 132 dyads CGs: relatives (55% female; 45% male; adult children, in-laws, nieces or nephews, or grandchildren), mean age = 44.46 (10.90) CRs: relatives (78% female; 22% male), mean age = 76.59 (7)	Hearing difficulties	CRs and CGs: Socio-demographics CRs: Objective caregiving stressors (MMSE; ADL; BEA; GHABP; GDS-15) CGs: Subjective stressors (CADI; MI)	CGs and CRs: Family relationships (FRI)	CRs’ health conditions and CGs’ negative reactions to care caused detrimental changes in family relationships over time for both the dyad members *[R^2^* = *0.20, F(6, 99)* = *4.14, p* < *0.001]*.	Associations between CR and CG intrapersonal variables (CR: health conditions; CG: reactions to care) and CG-CR relational wellbeing (quality of relationship)
Rand et al. (2017); United Kingdom	Cross-sectional survey-study	To explore the interdependence of three care related Quality of Life (QoL) attributes: control over daily life, social participation and occupation within the caregiving relationship	*N* = 264 dyads CGs: relatives (46% male), *n* = 135 (45.3%) ≥ 65 years^a^ CRs: relatives (41,6% male), *n* = 168 (56.4%) ≥ 65 years^a^	Aging	CGs and CRs: Socio-demographics Household finances Self-rated health Satisfaction with services Social Care context (ASCS; SACE; items from the Survey of Carers in Households) CRs: Activities of Daily Living scale (I/ADLs)	CRs and CGs: Quality of life dimensions; control over daily life, social participation, occupation (ASCOT)	1. Higher level of CRs’ long-term needs, lower satisfaction with services and their older age were associated with lower ratings of CGs’ control: number of I/ADLs *(B* = *−0.145 (0.048), p ≤ 0.01)* and satisfaction with services: *(B* = *0.652 (0.317), p ≤ 0.05)*; and of CGs’ social participation: aged 65 + years *(B* = *0.775 (0.317), p ≤ 0.05)* and number of I/ADLs *(B* = *−0.108 (0.044), p ≤ 0.05)*. 2. CGs’ difficulties with household finances were significantly associated with lower ratings of control over daily life by CRs *(B* = *−0.751 (0.350), p ≤ 0.05)*. 3. Moderate correlations were found between CGs’ and CRs’ control over daily life *(r* = *0.32, p ≤ 0.01)*.	1. Associations between CR intrapersonal variables (ADL, age, satisfaction with services) and CG emotional wellbeing (quality of life) 2. Associations between CG intrapersonal variables (difficulties with finances) and CR emotional wellbeing (quality of life) 3. CG and CR showed correlations in control over daily life
Raveis et al. (2000); United States	Cross-sectional survey study	To investigate individual and situational factors as predictors of psychological distress of adult daughter caregivers	*N* = 164 dyads CGs: adult daughters, mean age = 38.6 (7.6) CRs: parents (42% men), mean age = 68.8 (6.1)	Cancer	CGs Socio-demographics Physical health Living arrangements Caregiving experience (items derived from a pool of items assessing caregiver reactions) Interpersonal support (ISEL) CRs Socio-demographics Clinical information Health outcomes (RAND Health Survey—1 item)	CGs Anxiety (STAI-S)	CRs’ characteristics (e.g., advanced stage of the disease, increased time from diagnosis, poorer general health) were associated with the level of anxiety in caregiving daughters *[R^2^* = *0.098, F(6, 131)* = *2.370, p* = *0.033]*. Greater caregivers’ perceived availability of social support (i.e., facilitators) was associated with less caregiver’s level of anxiety *[change in R^2^* = *0.173, F(6, 121)* = *5.618, p* = *0.001]*.	Associations between CR/CG intrapersonal variables (CR: health conditions; CG: perceived availability of social support from others) and CG emotional wellbeing (anxiety)
Wu et al. (2021); China	Cross-sectional survey study (From the Chinese Longitudinal Healthy Longevity Survey)	To describe the characteristics of older adult care recipients and their adult-child caregivers, and to examine whether these characteristics are associated with caregiver burden	*N* = 168 dyads CGs: adult children (64.3% were male), mean age = 56.7 (7.3) CRs: older parents (56.6% female), mean age = 92.3 (9.5)	Aging	CGs and CRs: Socio-demographics Care related variables CGs: Filial Piety Scale CRs: Activities of daily living (ADL) Positive emotions (3 items created *ad hoc* for the study)	CGs: Caregiver burden (ZBI)	CRs positive emotion status [*B* = *−0.227 (−0.412, −0.042), p ≤ 0.01]*, multiple chronic conditions [*B* = *0.513 (0.081, 0.945), p ≤ 0.01]*, and CG intensity of care [*B* = *0.225 (0.061, 0.389), p ≤ 0.01]*, were associated with caregiver burden.	Associations between CR intrapersonal variables (CR positive emotions and multiple chronic conditions) and CG emotional wellbeing (burden)

*^a^Missing information on mean age. CG, caregiver; CR, care recipient; M, mean; SD, standard deviation; B, beta coefficients; CES-D, Center for Epidemiologic Studies Depression Scale; STAI-S, State Anxiety Scale of the State-Trait Anxiety Inventory; ISEL, Interpersonal Support Evaluation List; ASCS, Adult Social Care Survey; SACE, Survey of Adult Carers in England; ASCOT, Adult Social Care Outcomes Toolkit; POMS-SF, Profile of Mood States-Short Form; MOS-SF, Medical Outcomes Study 36-Item; BEA, Better Ear Average; GHABP, Glasgow Hearing Aid Benefit Profile; ADL scale, Activities of daily living; CADI, Carers’ Assessment of Difficulties Index; MMSE, Mini-Mental State Examination; GDS-15, Geriatric Depression Scale; FRI, Family Environment Scales; MIS, Lewis Mutuality and Interpersonal Sensitivity Scale; FHI, Family Hardiness Index; ISOC, Interpersonal Sense of Control Scale; SDPT, Self-Dyadic Perspective-Taking Scale; NEO FFI-Form S Scale, Personality Inventory; ISQ, Inner Strength Questionnaire; CAS-R, The Control Attitude Scale-Revised; CAS-Family, The Control Attitude Scale-Family; SF-36, Medical Outcomes Study 36-Item Short Form Health Survey; ZBI, Zarit Burden Inventory.*

**TABLE 2:** Criteria from the Mixed Methods Appraisal Tool.

Included studies	1.1	1.2	1.3	1.4	1.5	4.1	4.2	4.3	4.4	4.5	5.1	5.2	5.3	5.4	5.5.	Total quality score
Bouldin et al. (2019)						1	1	1	0	1						3/5
Heid et al. (2016)	1	1	1	1	1											3/5
Hollis-Sawyer (2001)											0	1	1	1	1	4/5
Kim et al. (2008)						1	1	1	0	1						4/5
Martini et al. (2001)						0	0	1	0	0						1/5
Romano et al. (2020)						1	1	1	0	1						4/5
Shawler et al. (2018)						1	0	1	0	1						3/5
Gilligan et al. (2017)						1	1	0	1	0						3/5
Jones and Morris (2013)	1	1	1	1	1											5/5
Katapodi et al. (2018)						1	1	1	0	1						4/5
Knussen et al. (2005)						0	0	1	0	1						2/5
Rand et al. (2017)						1	1	1	1	1						5/5
Raveis et al. (2000)						0	1	1	1	1						4/5
Wu et al. (2021)						1	1	1	0	0						3/5

*0 = No; Cannot tell; 1 = Yes; Qualitative studies: 1.1. Is the qualitative approach appropriate to answer the research question? 1.2. Are the qualitative data collection methods adequate to address the research question? 1.3. Are the findings adequately derived from the data? 1.4. Is the interpretation of results sufficiently substantiated by data? 1.5. Is there coherence between qualitative data sources, collection, analysis, and interpretation? Quantitative descriptive studies: 4.1. Is the sampling strategy relevant to address the research question? 4.2. Is the sample representative of the target population? 4.3. Are the measurements appropriate? 4.4. Is the risk of non-response bias low? 4.5. Is the statistical analysis appropriate to answer the research question? Mixed methods studies: 5.1. Is there an adequate rationale for using a mixed methods design to address the research question? 5.2. Are the different components of the study effectively integrated to answer the research question? 5.3. Are the outputs of the integration of qualitative and quantitative components adequately interpreted? 5.4. Are divergences and inconsistencies between quantitative and qualitative results adequately addressed? 5.5. Do the different components of the study adhere to the quality criteria of each tradition of the methods involved?*

#### Associations Between Intra/Interpersonal Variables and Wellbeing in Dyads

Seven studies (7/14; 50%) examined intrapersonal and interpersonal variables associated with emotional and relational wellbeing (e.g., quality of life, caregiver burden, depressive symptoms, relationship quality) of caregiver and care recipient dyads. *Intrapersonal* variables include individual-difference factors, such as psychological distress, attachment orientations, personality characteristics, and intellectual abilities of both the dyad members. *Interpersonal* variables include relationship-oriented factors such as perspective-taking skills, perceived strength of the caregiving relationship, communication patterns between caregivers and care recipients, and dyadic behavioral responses to goal differences in care. Given the heterogeneity of the psychological variables investigated in the included studies, results are presented below grouping first all *intrapersonal* variables and next *interpersonal* variables. Overall, regardless of whether the investigated psychological variables pertained to the individual (i.e., intrapersonal variables) or to the relationship (i.e., interpersonal variables), psychological variables were found to be associated with different levels of dyad members’ emotional and relational wellbeing in all studies.

Findings from a number of studies suggested interdependence in dyad members’ wellbeing by means of associations between care recipients’ intrapersonal variables and caregivers’ wellbeing (Hollis-Sawyer, 2001; Kim et al., 2008; Romano et al., 2020). For example, in a cross-sectional observational study, higher levels of psychological distress in care recipients were significantly related to lower levels of quality of life in caregiving daughters, but not vice versa (Kim et al., 2008). In a similar vein, fewer positive emotions and more insecure attachment orientations in older care recipients were associated with higher burden in adult-child caregivers (Romano et al., 2020). Only in one cross-sectional mixed method study, evidence for interdependence suggested that both caregivers’ and care recipients’ intrapersonal variables such as personality characteristics (e.g., caregiver’s neuroticism and care recipient’s openness to experience) and fluid intellectual ability (i.e., the ability to solve problems under novel situations) were significantly related to higher relationship quality in both dyad members (Hollis-Sawyer, 2001).

Similarly, findings of a number of studies suggested interdependence in dyad members’ wellbeing by means of associations with caregivers’ and care recipients’ interpersonal variables. All interpersonal variables examined relationship processes and highlighted reciprocity between caregivers and care recipients (e.g., understanding reciprocal needs, being connected with the other one, collaborative interactions, congruences in care goals) resulting in enhanced emotional and relational wellbeing (Martini et al., 2001; Heid et al., 2016; Shawler et al., 2018; Bouldin et al., 2019). For example, in a cross-sectional study, perspective-taking skills such as stronger interpersonal control (i.e., showing emotional control in reciprocal interactions and refraining from manipulative behaviors) and in-depth understanding of reciprocal thoughts and feelings (i.e., perceptual accuracy) were associated with greater satisfaction in the relationship for both the caregiver and the care recipient. More specifically, mothers who understood their daughters’ costs of helping and motives for helping had more satisfied daughters. Similarly, daughters who understood their mothers’ care needs and costs of being helped had more satisfied mothers (Martini et al., 2001). Perceived strength of the caregiving relationship in a longitudinal study, was found to be associated with dyad members’ physical and mental wellbeing over time, with higher perceived strength of the relationship at baseline, in both caregivers (i.e., daughters) and care recipients (i.e., mothers with hypertension), associated with higher overall health related quality of life at 6 months (Shawler et al., 2018). Communication patterns were also found to be associated with different levels of care recipients’ depressive symptoms reported by both caregivers (i.e., daughters, sons, siblings, or friends) and care recipients (Bouldin et al., 2019), with caregiving dyads defined as “collaborative” (i.e., frequent positive interactions between caregivers and care recipients) experiencing fewer depressive symptoms. Conversely, caregiving dyads defined as “avoidant” (i.e., avoided conversations related to illness), “distant” (i.e., not in frequent contact), or “antagonist” (i.e., frequent unpleasant and conflictual contact) reported more depressive symptoms in care recipients in comparison to other dyads. Lastly, a qualitative study highlighted how caregiving daughters and older parent care recipients manage interpersonal conflicts when there are differences in care goals (e.g., when daughters define what is the best care for their older parents but this is not in line with their older parents’ preferences). Dyad members described two different scenarios: (1) the caregiver may reason with the care recipient (e.g., due to different perceptions on where the parent should live, temperature of the room, social activities of the parent), and the care recipient accepts the caregivers’ requests by means of being more passive and “letting go” of these requests. In this situation, the caregivers’ goal is met and caregivers’ wellbeing preserved; or (2) care recipients may continue to act on their goal with active attempts to persist in their behavior, or hold discordant opinions from the caregiver. Subsequently, caregivers may gradually “let go” of the care recipients’ request to avoid conflicts. In this case, the care recipients’ goal is met. In either scenario, findings illustrate the difficulties adult daughters and older parents may experience when navigating care issues and how incongruences may negatively affect their reciprocal wellbeing and the caregiving situation (Heid et al., 2016).

#### Associations Between Care Context Variables and Wellbeing in Dyads

Seven studies (7/14; 50%) investigated whether care context variables were associated with levels of emotional and relational wellbeing (e.g., quality of life, anxiety, caregiver burden, relationship quality, and reciprocity) of caregiving dyads. Overall, findings suggested interdependence in dyad members’ wellbeing due to shared care and situational factors. Results are synthesized below presenting first, care context variables including care recipients’ health conditions and physical impairments associated with different levels of caregivers’ emotional and relational wellbeing; and second, care context variables defined as situational factors such as perceptions of social support associated with dyad members’ wellbeing. Both care recipients’ health condition and situational factors were found to be associated with emotional and relational wellbeing of dyad members.

Findings of a number of studies suggested interdependence by means of associations between care recipients’ health care needs, older age, and presence of multiple chronic health conditions, and lower emotional wellbeing in adult-child caregivers, in terms of lower ratings of control over daily life and social participation (Rand et al., 2017), higher levels of burden (Wu et al., 2021), and higher levels of anxiety (Raveis et al., 2000). Moreover, care recipients’ physical impairments were found to be associated with dyad members’ relational wellbeing in several ways. For instance, in one qualitative study, the clinical condition of young adult-child stroke survivors was associated with both positive and negative outcomes in the quality of the caregiving relationship. Both caregivers and care recipients, reported consequences (e.g., negotiating independence vs. dependence and changed relationships) and intense emotions (e.g., emotional turmoil) associated with difficulties adjusting to the caregiving role. Some experienced a sense of growth and improved communication between caregivers and care recipients. Conversely, others reported a detrimental impact of the disease on their relationships and restrictions in many areas of their intimate and social life (Jones and Morris, 2013). In line with the detrimental effects of the care recipients’ health condition on the caregiving relationship, in a longitudinal quantitative study, older care recipients’ hearing disabilities, cognitive impairments, and dependence in daily activities, contributed to relationship difficulties between caregivers and care recipients (i.e., compromised dyadic interactions) over time (Knussen et al., 2005).

With regard to perceptions of social support in the care context, care recipients’ higher satisfaction with the use of social services (e.g., community-based care) was significantly correlated with higher caregivers’ emotional wellbeing (i.e., higher control over daily life) (Rand et al., 2017) and caregivers’ perceptions of adequate availability of social support from others (e.g., family or friends) was found to be related with lower levels of self-reported anxiety (Raveis et al., 2000). On the other hand, when caregivers required more formal support due to difficulties with household finances, care recipients reported less control in their daily life, and lower quality of life (Rand et al., 2017). Lastly, although not statistically significant, some other care context variables were examined as indicators of caregiving dyads interdependence, such as caregivers’ self-rated health and intensity of care (i.e., 50 + hours of care per week) and care recipients’ problems with household finances (Rand et al., 2017). In another longitudinal quantitative study with families comprising triads of adult children with a number of severe health conditions, adult children without health conditions, and caregiving mothers, the intergenerational exchange of support was affected (Gilligan et al., 2017). For example, caregiving mothers were more likely to provide expressive and instrumental support to their adult children with serious health conditions, rather than to the healthy adult children, who were considered as “secondary caregivers” for their siblings. Healthy adult children were found to provide more expressive support to both their mothers and their ill siblings than they received, resulting in a lack of reciprocity in the adult-child and mother relationship.

#### Patterns of Covariation Between Caregivers’ and Care Recipients’ Wellbeing

In four studies, interdependence in dyad members’ wellbeing was also identified as patterns of covariation between caregivers’ and care recipients’ outcomes. Results are synthesized below reporting on correlations (e.g., psychological distress and quality of life) and congruent perspectives (e.g., ratings on care recipients’ depressive symptoms and responses to care) between caregivers and care recipients possibly impacting their emotional and relational wellbeing.

Findings suggested interdependence by means of correlations between dyad members’ wellbeing (Kim et al., 2008; Rand et al., 2017) and congruences on perceptions of depressive symptoms in the care recipients (Bouldin et al., 2019), and patterns of responses to goal differences in care (Heid et al., 2016). For example, in a longitudinal study, adult-daughter caregivers’ psychological distress was strongly positively associated with older mother cancer care recipients’ psychological distress, from the earlier phase of the illness to approximately 2 years post-diagnosis (Kim et al., 2008). Significant moderate positive correlations were also found in the quality of life of caregivers and care recipients with long-term care needs (Rand et al., 2017). Another study indicated that caregivers’ ratings of the presence of care recipient depression were moderately correlated to care recipients’ own self-assessments in those dyads that were characterized either by frequent positive interactions (i.e., collaborative dyads) or fewer negative interactions (i.e., distant dyads) (Bouldin et al., 2019). Lastly, in a qualitative study, adult-child caregivers and older care recipients who presented similar patterns of responses to care (e.g., both dyad members avoiding conflicts or brainstorming new solutions to goal differences in care), were likely to be the most satisfied and less conflictual. Congruent perspectives on the caregiving situation were found to prevent tense interactions between caregivers and care recipients, resulting in fewer negative implications for emotional and relational outcomes of dyads (Heid et al., 2016).

## Discussion

Findings synthesized in the current review suggest that there is dyadic interdependence in the emotional and relational wellbeing of non-spousal caregiving dyads (e.g., mainly among adult children and parents, but also siblings, other relatives, or friends). Evidence for dyadic interdependence of non-spousal dyads was found in accordance with interdependence theory (Rusbult and Van Lange, 2003; Van Lange and Rusbult, 2012). Indeed, interdependence was found by investigating the *level of dependence* between dyad members, that is the impact of each dyad member on the wellbeing of the other one; the *structure* that is the shared context of the caregiving situation; and the *covariation of interests*, as patterns of covariation between caregivers’ and care recipients’ outcomes.

In line with the three dimensions of the interdependence theory mentioned above, findings from research on non-spousal caregiving dyads supported dyadic interdependence. On a first level of evidence for interdependence, research on non-spousal caregiving dyads has shown that good psychological functioning of care recipients, and specific personality traits and intellectual abilities of both caregivers and care recipients (i.e., intrapersonal variables) might impact reciprocal emotional and relational wellbeing of dyad members (Hollis-Sawyer, 2001; Kim et al., 2008; Romano et al., 2020). Moreover, one element of dyadic interdependence strongly reported in a number of studies synthesized in this review was relationship processes (i.e., interpersonal variables) such as communication patterns and dyadic behavioral responses to care may be associated with different levels of dyad members’ wellbeing (e.g., quality of life, caregiver burden, depressive symptoms, and relationship quality) (Martini et al., 2001; Heid et al., 2016; Shawler et al., 2018; Bouldin et al., 2019). In line with studies investigating relationship processes of spouses dealing with various illnesses (Laurenceau et al., 1998; Lepore, 2004; Manne and Badr, 2008; Hagedoorn et al., 2011b), our findings suggest that, for example, a shared perception of the quality of the caregiving relationship as well as collaboration, open communication, and positive dyadic responses to care might increase wellbeing outcomes for both members of non-spousal caregiving dyads (Heid et al., 2016; Shawler et al., 2018; Bouldin et al., 2019). Furthermore, findings are consistent with previous research on spousal caregiving dyads showing that life-threatening events (e.g., chronic illnesses) cause high levels of stress for each partner and significant challenges for the relationship as well (Hagedoorn et al., 2008; Dorros et al., 2010; Fife et al., 2013). While the experience of individuals is certainly important, some elements of caregiving are ineluctably relational, and research acknowledging the interconnectedness of caregivers and care recipients is needed in order to provide a more sophisticated analysis of interactions, such as decision making, communication, and dyadic coping in diverse illness contexts (Revenson et al., 2016). Therefore, a dyadic approach in caregiving allows a more accurate assessment of the factors determining dyad members’ wellbeing.

On a second level of evidence for interdependence, research on non-spousal caregiving dyads has confirmed the crucial role of the care context (i.e., the same caregiving/family context) for both dyad members’ wellbeing. A number of care context and situational variables (e.g., care recipient’s health conditions, caregiving tasks, and the broader social environment) were found to be associated with emotional and relational wellbeing of the dyad. Indeed, care recipients’ poor physical health was generally related to lower caregivers’ quality of life (i.e., mental and physical health), regardless of disease type (Raveis et al., 2000; Knussen et al., 2005; Jones and Morris, 2013; Gilligan et al., 2017; Rand et al., 2017; Katapodi et al., 2018; Wu et al., 2021). This pattern of results is consistent with previous literature concerning caregivers experiencing a loss of autonomy and life satisfaction due to care recipients’ care needs (Borg et al., 2006). Traditionally studies identified various characteristics of the care recipient (e.g., advanced and terminal diseases, cognitive impairments, behavioral problems) and of the provision of care (e.g., higher number of hours spent in caregiving, co-residence with the care recipient) as predictors of caregiver burden (Adelman et al., 2014). Our findings, suggest that the broader social environment (i.e., perception and evaluation of social support available) seems to play a crucial role in defining wellbeing for both caregivers and their care recipients (Raveis et al., 2000; Rand et al., 2017).

Lastly, on a third level of evidence for interdependence, dyad members’ wellbeing was found to be strongly correlated (e.g., similar level of psychological distress and quality of life) and when both caregivers and care recipients reported to have congruent perspectives on the caregiving situation (e.g., similar ratings of care recipients’ depressive symptoms and congruent responses to goal differences in care) they also reported enhanced wellbeing (Kim et al., 2008; Heid et al., 2016; Rand et al., 2017; Bouldin et al., 2019). These findings are in line with a meta-analysis which found similarities between the mental health of adult-child caregivers and their cancer patients. Patterns of covariation in close relationships suggest that one person’s affect could “cross-over” to the other person, resulting in an “interpersonal emotion transfer” (Hodges et al., 2005). It is possible that people “catch” the intense emotional states of others with whom they interact and then acquire their similar states and behaviors (Hatfield et al., 1993). In addition, congruent perspectives might also depend on the shared context of the caregiving situation where both dyad members respond to and interact while providing and receiving care. Sharing the same environment (e.g., same house, frequent interactions, care tasks) is considered as a facilitating factor of interdependence. A number of studies within different illness contexts (e.g., cancer, stroke, and dementia) with spouses have demonstrated strong correlations on ratings of quality of life, care recipients’ symptoms and distress (Stanton et al., 2007; Mitchell et al., 2014). Further research should investigate whether covariation in dyad members’ outcomes depends on crossover processes or merely reflects reactions to a shared psychosocial environment.

### Study Gaps Identified

Combined, these studies suggest various caregiving difficulties should be addressed at a dyadic level. Findings from this systematic review demonstrate the concept of “linked lives” in non-spousal dyads, referring to how the experiences of individuals in interdependent dyadic relationships affect not only the individual but also the other family member (Gilligan et al., 2017). There is a growing interest in investigating dyadic interdependence within non-spousal dyads. However, currently there is a dearth of literature concerning the dyadic perspective in non-spousal dyads, as shown by the small number of included studies (*n* = 14), and findings are heterogeneous underscoring the complexity and multidimensionality of caregiving experiences.

Further research is needed in this area. First, future studies should differentiate between caregiver subgroups (e.g., spousal vs. non-spousal dyads) in order to provide a clearer understanding of dynamics within different relationship type dyads (Braun et al., 2009). In our review, some relevant studies examining interdependence were excluded due to reporting data from mixed samples comprised of both spousal and non-spousal caregivers, without presenting findings separately for these two different groups (Lyons et al., 2002; Li et al., 2014; Dellafiore et al., 2019). Consequently, data relevant to our review has been omitted. Due to the unique nature of family relationships, many differences may exist between spousal and non-spousal relationships (e.g., socio demographic variables, gender differences, filial obligation, intensity of care, and living arrangements) (Pinquart and Sörensen, 2011). Moreover, even if we decided to disentangle spousal dyads from non-spousal ones, it is worth mentioning that among non-spousal dyads there might still exist substantial differences (e.g., adult children, siblings, friends) which should be considered in future dyadic caregiving studies.

Overall, our findings highlight a need to incorporate the dyadic perspective in caregiving research, whilst also addressing the broader care context. The present review highlighted that studies tend to either examine first level of evidence (i.e., intrapersonal and interpersonal variables), or studies examine the second level of evidence (i.e., care context variables). As such, psychological and relational factors are often explored in dyadic studies without addressing the broader care context and, on the other hand, other caregiving studies focus on caregiving factors only (e.g., care recipients’ health conditions, hours and types of caregiving tasks), neglecting psychological and relational factors (e.g., communication and dyadic coping). Further research should combine these perspectives (Revenson et al., 2016).

The majority of included studies focused on interpersonal variables (i.e., relationship processes), in line with the existing spousal literature. Certain aspects of communication, such as mutual constructive communication, self-disclosure, partner responsiveness, have been found to be associated with higher levels of intimacy and relationship satisfaction in various couples dealing with illness, especially cancer (Manne and Badr, 2008; Manne et al., 2010). However, emotional disclosure was not found to reduce distress in similar spousal dyads dealing with cancer (Porter et al., 2005; Hagedoorn et al., 2011b). In the non-spousal literature, there is a paucity of studies investigating which interpersonal and relationship processes may be harmful or beneficial for non-spouses’ wellbeing. Some issues examined in the spousal literature (e.g., intimacy processes) deserve further attention in other caregiver-care recipient dyads. Future research may wish to extend the dyadic literature by developing a new theoretical framework suitable for dyads other than spouses. For example, the developmental-contextual model of dyadic coping, although developed for spouses, is applicable to other dyads: with adolescents and their parents, but also adult children and elderly parents (Berg and Upchurch, 2007; Berg et al., 2009).

Another valuable avenue of future research in this area is the design and development of effective dyadic interventions tailored for both the caregiver and the care recipient in close, but non-romantic, relationships (Badr, 2017). Evidence of interdependence may provide a guide for researchers and health care practitioners to understand to what extent dyad members are involved together in coping with illness or aging. It is therefore important to identify in which interpersonal contexts dyadic interventions can support both dyad members, given if dyad member were to become distressed, it is more than likely that the other dyad member will also. When designing interventions to improve wellbeing or to promote dyadic coping behaviors, there is some evidence that accounting for dyadic-level influences is more successful compared to limiting the focus to the individual (Crepaz et al., 2015). Identification of those factors, which contribute to affect caregivers’ and care recipients’ mutual wellbeing is important for future policy and practice development. For example, when care recipients’ needs and dependence on caregivers increases, caregivers’ ability to maintain a high level of care may be affected, resulting in lower wellbeing for both dyad members. Therefore, policy underpinning support for and psychosocial needs of caregivers who provide care to an older parent, a sibling or any other relative or friend, may consider to improve both the dyad members’ outcomes, thus potentially reducing economic costs related to the use of health services.

Lastly, on a methodological level, powerful dyadic analytic techniques (e.g., actor-partner interdependence model; APIM) have been extensively used in spousal research and they may be integrated also into future non-spousal caregiving research (Kenny et al., 2006). In our included studies, only four studies accounted for non-independence using the APIM model which allows testing of the actor (i.e., intrapersonal) and partner (i.e., interpersonal) effects simultaneously and permits to explain the covariance between the outcomes of both the members of the dyad. The APIM model, either using multilevel modeling or structural equation modeling, integrates a conceptual view of interdependence in two-person relationships with the appropriate statistical techniques for measuring and testing it (Cook and Kenny, 2005).

### Limitations and Strengths of This Review

It is important to acknowledge that our review has several limitations: the small number of included studies rendered a statistical meta-analysis impossible to perform; moreover, the 14 studies used diverse variables to measure the constructs of interest and individual study sample sizes were sometimes small. The cross-sectional design of a number of included studies meant that the directionality of associations and influences was not always clear, and more longitudinal studies are needed. Other limitations may be due to the exclusion of non-English studies. Furthermore, the majority of studies were conducted in United States and therefore findings might not be generalizable to other countries and cultures. Further research is needed to investigate whether dyadic interdependence is influenced by socio-contextual and cultural factors. Finally, due to the small number of studies, findings where synthesized across a number of different non-spousal dyad types including adult children taking care of their parents, but also parents taking care of their adult children, siblings, other relatives, or friends. Although these dyads are all non-spousal, many similarities and differences may still exist in the nature of the relationship types (e.g., patterns of support may vary depending on family structures, norms, and values) and in the consequences reported (e.g., adult children may report more negative consequences on their wellbeing than friends or non-relatives). Future research should avoid the risk of treating caregivers as a homogeneous group by taking into account differences in the relational and social context of caregiving.

The strengths of our review are reflected, first, in including studies that considered the perspective of both dyad members. Dyadic designs allow researchers to test for interdependence both on a theoretical (i.e., caregiving as a dyadic process) and methodological (e.g., non-independence of dyadic data) level (Cook and Kenny, 2005). Another strength of the review is the adoption of a theoretical framework (i.e., interdependence theory) to guide the review process and systematically synthesize the existing literature on interdependence within non-spousal dyads. Moreover, most of the included studies were of high quality and used validated and standardized measures. Lastly, another strength was to pre-register the review in PROSPERO before conducting it (Ioannidis, 2014).

## Conclusion

Evidence supporting dyadic interdependence among non-spousal caregiving dyads informs a growing understanding of mutual influences among dyad members beyond the traditional spousal research. Identification of the different ways wellbeing of one dyad member may depend on the other dyad member and vice versa (i.e., dyadic interdependence) has important clinical implications for the development of interventions aimed to improve the outcomes of both caregivers and care recipients. However, the current review identified limited research on interdependence in non-spousal caregiving field, suggesting many potential future avenues of research on interpersonal and relationship processes among non-spousal caregiving dyads. The review did identify some levels of evidence for interdependence between dyad members, which warrants further investigation. Taking into account the type of relationship between caregiver and care recipient (e.g., being a spouse/partner or an adult child) provides an opportunity to examine whether influences of each individual’s functioning on the wellbeing of their companion may also depend on the context of interpersonal relationships and the broader psychosocial environment. In conclusion, understanding the nature and the processes of dyadic relationships offers valuable insights into caregiving as a relational phenomenon also within non-spousal dyads.

## Data Availability Statement

The original contributions presented in the study are included in the article/Supplementary Material, further inquiries can be directed to the corresponding author/s.

## Author Contributions

GF created the search string with the help of the UMCG librarian and undertook the search in the electronic database for the relevant articles. GF and SD screened the relevant article for eligibility based on the inclusion criteria. GF, SD, JW, and MH conducted the narrative analysis to synthesize the data for both qualitative and quantitative studies. GF and MH drafted the article. SD and JW edited the subsequent drafts. All authors contributed to the article and approved the submitted version.

## Conflict of Interest

The authors declare that the research was conducted in the absence of any commercial or financial relationships that could be construed as a potential conflict of interest.

## Publisher’s Note

All claims expressed in this article are solely those of the authors and do not necessarily represent those of their affiliated organizations, or those of the publisher, the editors and the reviewers. Any product that may be evaluated in this article, or claim that may be made by its manufacturer, is not guaranteed or endorsed by the publisher.
