# Supplementary material for: Dyadic Interdependence in Non-spousal Caregiving Dyads’ Wellbeing: A Systematic Review
Source: Front Psychol. 2022 Apr 29;13:882389. doi: 10.3389/fpsyg.2022.882389 (PMC9102382; doi:10.3389/fpsyg.2022.882389)
Supplement: Supplementary file 2 [file Table_2.pdf]

S2 Supplementary Material

***PRESS Guideline* — Search Submission & Peer Review Assessment**

SEARCH SUBMISSION: THIS SECTION TO BE FILLED IN BY THE SEARCHER

Systematic Review Title:

Dyadic interdependence in non-spousal caregiving dyads' wellbeing: a systematic review

This search strategy is …

X	My PRIMARY (core) database strategy — First time submitting a strategy for search question and database
	My PRIMARY (core) strategy — Follow-up review NOT the first time submitting a strategy for search question and database. If this is a response to peer review, itemize the changes made to the review suggestions
	SECONDARY search strategy— First time submitting a strategy for search question and database
	SECONDARY search strategy — NOT the first time submitting a strategy for search question and database. If this is a response to peer review, itemize the changes made to the review suggestions

Database

PsycINFO, PUBMED, CINAHL

Interface

EBSCO and MEDLINE

Research Question

1. How many studies undertook dyadic perspective examining associations between intrapersonal, interpersonal, care context variables and individual and dyadic wellbeing in non-spousal caregiving dyads (e.g., adult children – parents, siblings etc.)?

2. Might these associations be considered as indicators of dyad members' interdependence within non-spousal caregiving dyads?

Aim

The purpose of the systematic review is to review studies that examined the interdependence between non-spousal caregiving dyad members (e.g., adult children – parents, siblings etc.). Interdependence was defined using Cook and Kenny's definition: "There is interdependence in a relationship when one person's emotion, cognition, or behaviour affects the emotion, cognition, or behaviour of a partner". (Cook & Kenny, 2005). Investigating interdependence of psychological and interpersonal processes in patient-caregiver dyad allows researchers to examine the influence of each individual's psychological functioning on the well-being of their companion, leading to the identification of connections between caregiver, care recipient and the illness context. Romantic couple's literature has found that relationships are inherently interdependent in nature – the outcomes that one partner experiences become deeply intertwined with another partner's outcomes over time and across context (Clark & Mills, 2011; Kelley & Thibaut, 1987; Rusbult & Van Lange, 2003). Mainly spousal patients and caregivers exhibit dyadic interdependence, which suggests that a patient's well-being directly impacts caregiver's well-being and vice versa (Segrin, Badger, & Harrington, 2012). In caregiving literature, increasing interest is given also to different type of caregiving dyads, such as adult-children parents and other family member. Therefore, the first objective is to focus on interdependence between non spousal patient-caregiver dyads and, secondly, to describe the intra/inter-personal factors (e.g., dyadic coping, interactions, mutuality, relationship/family functioning) between these caregivers and care recipients , in order to highlight which components have the greatest potential for effecting changes on caregivers' psychological wellbeing (e.g., burden, stress or distress, anxiety, depression, mood, positive outcomes, relationship satisfaction) in these specific non-spousal relationships.

PICO Format

(Outline the PICOs for your question — i.e., Patient, Intervention, Comparison, Outcome, and Study Design — as applicable)

**P**	Informal Caregivers and their care recipients.
**I**	Phenomenon of interest: interdependence and reciprocal interactions between dyad members (non spouses). Focus on intrapersonal, interpersonal and care context variables.

**C**	N/A
**O**	Caregiver outcomes (e.g., burden, stress or distress, anxiety, depression, mood, positive outcomes, relationship satisfaction) and correlations among dyad members' outcomes.
**S**	Qualitative (semi-structured interviews), Quantitative (longitudinal and cross-sectional design) and mixed method studies.

Inclusion Criteria

(List criteria such as age groups, study designs, etc., to be included)

- Adult (18 years or older) informal caregivers of adult care recipients (if the age of the CR is not specified, include) with chronic illness, disability or physical disability (e.g., cancer, heart disease, stroke, diabetes, dementia, COPD…). If not specified the type of illness, include.
- Care recipients are community dwelling and not receiving palliative care.
- Any type of relationship between CG-CR: adult children - parents, siblings. Do NOT INCLUDE spouses or romantic relationships.
- Among intra/inter-personal processes include: interactions and communication within caregiver and care recipient, dyadic coping, collaboration, social support provided or received, quality and type of relationship, relationship/family functioning, responsiveness, relational experiences.
- Studies with qualitative (semi-structured interviews), quantitative (longitudinal and cross-sectional) and mixed methods.
- Research report.
- Publication in English.
- Papers from all the years.

Exclusion Criteria

(List criteria such as study designs, date limits, etc., to be excluded)

- Studies with children (< 18 age).

- Spouses or romantic relationship between CG and CR.
- Intervention studies.
- Exclude studies with professional caregivers, medical staff, nurses (if the caregiver is not present).
- Grey literature: conference abstract, presentations, proceedings; regulatory data; unpublished trial data, government publications; reports (such as white papers, working papers, internal documentation); dissertations/thesis.
- Reviews and protocols.
- Case studies.

Was a search filter applied? No

Yes ■ No ■

If YES, which one(s) (e.g., Cochrane RCT filter, PubMed Clinical Queries filter)? Provide the source if this is a published filter. *[mandatory if YES to previous question — textbox]*

Other notes or comments you feel would be useful for the peer reviewer?

Based on the research question, the search string was finalized with the help of the Librarian of the Department of Health Psychology.

Please copy and paste your search strategy here, exactly as run, including the number of hits per line.

PsycINFO

(DE "Caregivers" OR TI caregiv*) AND (DE "Family" OR family OR parent* OR adult child* OR informal care)

AND

(DE "Interpersonal Interaction" OR DE "Assistance (Social Behavior)" OR DE "Collaboration" OR DE "Collective Behavior" OR DE "Conflict" OR DE "Cooperation" OR DE "Interdependence" OR DE "Interpersonal Communication" OR DE "Interpersonal Influences" OR DE "Psychological Distance" OR interpersonal OR dyadic OR communal coping OR collaborative coping OR responsiveness OR partner responses OR emotional expression OR mutuality OR family processes OR reciprocal OR congruence OR interdependence OR relationship change*)

AND

(DE "Major Depression" OR DE "Depression (Emotion)" OR DE "Stress" OR DE "Caregiver Burden" OR DE "Psychological Stress" OR DE "Distress" OR depress* OR distress OR stress OR burden OR DE "Well Being" OR DE "Quality of Life" OR quality of life OR wellbeing OR well being OR positive OR psychological adjustment OR relationship satisfaction OR anxiety OR mental health)

CINAHL:

(MH "Caregivers" OR TI caregiv*) AND (MH "Family+" OR family OR parent* OR adult child* OR informal care*)

AND

(MH "Interpersonal Relations+" OR interpersonal OR dyadic OR communal coping OR collaborative coping OR responsiveness OR partner responses OR emotional expression OR mutuality OR family processes OR reciprocal OR congruence OR interdependence OR relationship change*)

AND

(MH "Depression" OR MH "Stress, Psychological+" OR MH "Stress+") OR (depress* OR distress OR stress OR burden OR MH "Psychological Well-Being" OR MH "Quality of Life" OR quality of life OR wellbeing OR well being OR positive OR psychological adjustment OR relationship satisfaction OR anxiety OR mental health)

Pubmed:

("Caregivers"[Mesh] OR caregiv*[ti]) AND ("Family"[Mesh] OR family[tiab] OR parent*[tiab] OR adult child*[tiab] OR informal care[tiab])

AND

("Interpersonal Relations"[Mesh] OR interpersonal[tiab] OR dyadic[tiab] OR communal coping[tiab] OR collaborative coping[tiab] OR responsiveness[tiab] OR partner responses[tiab] OR emotional expression[tiab] OR mutuality[tiab] OR family processes[tiab] OR reciprocal[tiab] OR congruence[tiab] OR interdependence[tiab] OR relationship change*[tiab])

AND

(“Depression"[Mesh] OR "Stress, Psychological"[Mesh] OR "Quality of Life"[Mesh] OR depress*[tiab] OR distress[tiab] OR stress[tiab] OR burden[tiab] OR quality of life[tiab] OR wellbeing[tiab] OR well being[tiab] OR positive[tiab] OR psychological adjustment[tiab] OR relationship satisfaction[tiab] OR anxiety[tiab] OR mental health[tiab]
